# Insurance Bill Developments

**Published:** 1911-08-12

**Authors:** 


### INSURANCE BILL DEVELOPMENTS.

(From a Correspondent.)

The newspapers might justifiably adopt a new heading—"The Insurance Bill from Day to Day." For each day's discussion of the measure shows the adoption of one suggestion or the omission of some proposal, until the only thing certain about it is that when it becomes law it will be a very different Bill from what it was when it was first drafted. Mr. Lloyd George is now willing to concede the point that an insured woman, the wife of an insured man, should receive sick-pay during the time of her confinement as well as the 30s. maternity benefit. Of course, this does not settle the claims of the woman whose husband is not insured, because he admits that the special maternity benefit is to come from the men's fund. The situation stands thus: the non-insured wife of an insured man gets 30s. maternity benefit; the insured wife of a non-insured man gets 7s. 6d. a week (equal to 30s. for the month) maternity benefit; the insured wife of an insured man gets 30s. maternity benefit from her husband's fund and 7s. 6d. a week from her own. This seems to us to be a very fair equivalence of investment and gain; but there are not lacking critics who would give the double benefit to every mother, whether or not her husband is contributing. It is very certain that if this be granted, it will in turn arouse criticism from men who contribute week by week to the Insurance Fund, but for many years require nothing for themselves, but not infrequent grants towards the cost of an addition to their families. They may reasonably grumble if they have thus to contribute to the cost of the lazy and the incompetent, and, in fact, if maternity benefit be given without regard to contributions, the so-called insurance will be only Poor-law relief under another and a prettier name. If maternity *quâ* maternity is to be endowed, let it be done frankly, and not under the pretext of a contributory scheme.

Mr. Lloyd George promises also to withdraw the sub-section which prevents insured persons receiving sickness or disablement pay during any period when he was provided with board and lodging by his employer. This will remove an obvious objection to the Bill and will doubtless give great satisfaction. But, as we have already pointed out, many of the class who would under the sub-section have been thus differentiated against, will receive treatment in hospitals, and it is natural that hospital authorities are beginning to ask how the Bill will affect them. Judging by the opinions expressed by many hospital officials, the effect of the Bill will be to add to their responsibilities while lessening their income. Of the latter fact there can be no doubt. What people give in charity is what they can spare from their income beyond their own necessities. As this Bill must inevitably result in increased taxation, the margin at the disposal of the charitable will be diminished; they will have less to give to hospitals as to every other form of charity. It would seem reasonable if hospitals, as well as private doctors, were to receive some remuneration, even if it be of small amount, for their treatment of those who, though poor, can no longer be regarded as destitute. It would be better that this should be a charge on the patient rather than a payment from the State, so that the independence of the hospital may remain unimpaired. And this payment if begun, even on the small scale possible to those insured under this Bill, may lead to a development of those pay wards, with good treatment even for the poorest, but with luxuries proportioned to the sum demanded, which form an integral part of hospitals elsewhere.

Hospital authorities feel also that they would, as employers, be compelled to pay large sums for their employees, whom in any case they would have to treat, as they do now, within their own walls. It is also felt that, especially in the provinces, where working men contribute to hospitals more largely and more regularly than they do in London, they would feel themselves excused from this duty by their contributions to the Insurance Bill, while they would still come to the hospitals for treatment in any serious illness.

Among other grievances it is to be expected that there should be complaints from various trades. Thus both agricultural labourers and fishermen protest against having to pay as much as town-workers. The wages of the ploughman are lower than those of the artisan, and he has always been told, by way of consolation, that his work was healthier and his life longer than that of the town worker. From an old-age pension point of view this was eminently satisfactory, but the statement is looked upon with other eyes when it is a matter of sickness insurance. Why should they pay for other men's illness? It is true, as Mr. Lloyd George pointed out, that the man who is not cut off in his prime is the one who suffers from prolonged sickness in maturer years, but this argument is not likely to appeal to the hale and hearty man in middle life, and at least he will want to be assured that if he is ill after he reaches the age of seventy he will get his sickness benefit as well as his old-age pension. As for fishermen, not only is it true that when they are not cut off by accident they enjoy good health into old age, but the fact is also to be considered that in many cases they are part owners of their boat and tackle, and are paid in proportion to their holdings. This is not as true now as it was in a former day, but the conditions still obtain in many cases. That being so, who is the employer? The matter may be settled by making the co-operative fisherman pay both as employer and employee, but it is desirable that if this is to be the rule it should be made clear. It is a case when a few words in a Bill would save endless cost and annoyance in the law courts.

Among others who consider that their interests are not sufficiently regarded under the Bill are persons of foreign birth who are resident within the United Kingdom. A meeting has been held in the Great Assembly Hall, Mile End, to protest against the hardships inflicted on the Jewish Friendly Societies and the alien population by the proposed provisions of the Bill. Aliens, no matter how long they had lived in this country, would not profit under the Bill, although they would doubtless be compelled to pay the subsidy out of their wages, and certainly their employers would have to pay their share. The remedy is, of course, that they should be nationalised, but for various reasons this is often neglected, and thus the contributor gains nothing from his contributions, and there are some in whom the love of country is so strong that, even for their own interest, they will not denationalise and renationalise themselves. The demand that those at least should be admitted to the benefits of the Act who have been members of a friendly society for five years does not seem unreasonable.

Among other marvels which the Bill is hoped to achieve is the prevention of improvident marriages. Mr. C. Bathurst suggested that among those who should receive only reduced benefits in the case of illness should be "married persons if, in the opinion of the local Health Committee, physically or mentally unfit for marriage." Mr. Bathurst objects, as do many other people, to the marriage of epileptics, consumptives, and those who have no means of supporting a family. But until the law forbids such marriages and takes steps to prevent them, it does not seem practicable for any Health Committee to refuse the ordinary advantages offered under the Bill. The utmost that can be achieved is the limitation of benefit to 5s. a week for youths and 4s. for girls under the age of twenty-one; but it has been proposed, but this does not appear to apply to married couples, and there seems to be a general consensus of opinion that if boys or girls of eighteen have any member of their family dependent on them they should receive full money. Mr. Ramsay Macdonald would even have the age at which full benefits should be received in such a case reduced to sixteen, but it is questionable if it is wise to do anything to encourage the dependence of others upon such young persons. When such cases occur, they might surely be met by private or organised charity. It is a very serious matter if they should be so numerous that national aid must be called in.

